# Dengue haemorrhagic fever: an evaluation of host innates immune factors in hepatic lesions and their correlation with immunopathogenesis

**DOI:** 10.1590/S1678-9946202668021

**Published:** 2026-03-02

**Authors:** Carla Pagliari, Geovanna Menezes Marcoli, Luciane Kanashiro-Galo, Evandro Sobroza de Mello, Leda Viegas de Carvalho, Ricardo Penny, Juarez Simões Quaresma, Pedro Fernando da Costa Vasconcelos, Mirian Nacagami Sotto

**Affiliations:** 1 Universidade de São Paulo, Faculdade de Medicina, Departamento de Patologia, São Paulo, São Paulo, Brazil; 2 Instituto de Assistência Médica ao Servidor Público Estadual, Programa de Pós-Graduação em Ciências da Saúde, São Paulo, São Paulo, Brazil; 3 Fundação Champalimaud, Lisboa, Portugal; 4 Hospital Guilherme Álvaro, Serviço de Verificação de Óbito, Santos, São Paulo, Brazil; 5 Instituto Nacional de Ciência e Tecnologia para Viroses Emergentes e Reemergentes, Belém, Pará, Brazil

**Keywords:** Dengue, Liver, Innate immune response, IFN-I

## Abstract

Dengue fever challenges public health worldwide. The numerous factors associated with dengue fever severity and mortality risk include host characteristics such as patient age, comorbid conditions, previous dengue virus (DENV) infections, and biochemical biomarkers. Type I IFNs are essential cytokines in orchestrating innate and adaptive immune responses against viral invasion, and their regulation is mediated by IRF-2, which prevents excessive IFN expression. *In vitro* studies have shown DENV evasion strategies that affect IFN-I production but few have considered the *in-situ* interrelationship between IFN-I and the virus. This study aims to find elements of innate immunity that induce the anti-viral response and their correlation with the detected alterations in liver lesions. Liver specimens from individuals who died due to dengue were selected according to clinical and laboratory data and serological diagnosis. The specimens were subjected to histological and immunohistochemical evaluation of cells expressing IFN-I, RIG-1, IRF-2, and STING. Viral antigens were detected by an anti-DENV. A high number of cells expressed RIG-1 and IRF-2 when compared to IFN-I and STING. In severe cases of dengue, DENV may play a role in its pathogenesis with properties that induce non-effective immune responses. The virus can evade effective immune responses by impairing the early activation of innate immunity. This immune dysregulation may contribute to the progression of more severe manifestations and seems to play a role in the pathogenesis of hepatic involvement.

## INTRODUCTION

Dengue fever has become a pressing global challenge, as highlighted by the World Health Organization and the Pan American Health Organization. According to the Dengue Epidemiological Situation in the Region of the Americas, the area reported 4,294,752 suspected cases in epidemiological week 48 of 2025, resulting in a cumulative incidence of 409 cases por 100,000 population. (updated on December 1, 2025). The global dengue surveillance, according to data reported as of November 17, 2025, found 4,734,763 cases and 3,390 deaths from January to October 2025^1,2^.

In response, Brazil established its National Contingency Plan for Arboviruses in 2025 to control the spread of the virus (DENV) and reduce the risk of epidemics similar to that of 2024^3^.

Severe dengue can present plasma leakage that may lead to shock, fluid accumulation that causes respiratory distress, and severe bleeding or signs of organ dysfunction in the heart, lungs, kidneys, liver, or central nervous system.

Immunohistochemistry technique followed by histopathological analysis increasingly serves to evaluate tissue from post-mortem cases. These techniques have become essential for understanding the major complications due to DENV and the mechanisms by which the virus infects specific cells^3–5^.

Dengue has a deleterious and important effect on several organs, the most common being the liver. A case study of children who died from dengue haemorrhagic fever using immunohistochemistry found midzonal steatosis, hepatitis, and hepatocellular necrosis^6^.

Regarding innate immunity, experimental and *in* vitro tests have shown that DENV activates pattern recognition receptor pathways, such as cGAS-stimulator of interferon genes (STING) and RLR (retinoic acid-inducible gene I [RIG-I] and melanoma differentiation-associated protein 5 [MDA5]), which play crucial roles in innate immunity against viral infections. These mechanisms function as a bridge between the detection of viral pathogens and the activation of host defence mechanisms, including the production of IFN-α and IFN-β (type I interferons [IFN-I])^7^.

Type I IFNs are essential cytokines in orchestrating the innate and adaptive immune responses against viral invasion, and their regulation is mediated by IRF-2, which prevents excessive IFN increases. *In vitro* studies have shown DENV evasion strategies that affect IFN-I production. However, in tissues from DENV-induced lesions few studies have assessed the interaction between IFN-I and the virus^8–11^.

DENV‐infected hepatocytes produce IFN‐γ, an important cytokine to inhibit viral spread. It acts according to the expression of interferon‐stimulated genes, including IFITM1, IFTM3, IRF1, IRF7, and IFI6^12^.

These gene products protect host cells from viral invasion and antagonize host cell apoptosis. However, DENV can inhibit the effects of IFN by expressing NS5 and NS4B, compromising antiviral signalling pathways^13–17^.

The NS1 antigen can be detected in some organs, such as the liver and brain, with a high degree of impairment in the latter, as evinced by a high number of NK cells and cytokines IL1β, IL2, IL4, IL5, IL6, IL10, IL13, and IL15^18^. However, this disease affects multiple organ systems, the commonest being the liver. Starting from asymptomatic high transaminase levels to acute liver failure, dengue has all the properties of a hepatic illness^19^.

Under DENV high viral loads, hosts’ protective response may become imbalanced and overwhelmed, resulting in infection of more hepatocytes and extensive liver injury.

This study aims to find *in situ* elements of innate immunity that induce the anti-viral response and their correlation with the detected alterations in liver lesions.

## MATERIALS AND METHODS

### Patients, samples and diagnosis of dengue infection

In total, 20 liver specimens from individuals who died due to haemorrhagic dengue during the outbreak of 2010 were selected according to clinical and laboratory data and serological diagnoses from the archives of the Department of Pathology at the Hospital Guilherme Alvaro, in the Santos municipality, Sao Paulo State, Brazil.

### Sample analysis

The formalin-fixed, paraffin-embedded specimens were stained in haematoxylin-eosin for histological evaluation. Samples were hydrated in a series of ethanol grades. Endogenous peroxidase activity was blocked, and sections were incubated in an antigen retrieval solution at pH 9.0 for 20 min at 95 °C. Immunohistochemical staining was performed with the following primary antibodies, over-night at 4 °C: anti-IFN I (α/β), anti-RIG 1, anti-IRF 2, and anti-STING. A biotin-Free Polyvalent-HRP system was used to develop the reactions. Diaminobenzidine was used as the chromogen. The specifications of primary antibodies are listed in Table 1. All reactions were performed with positive and negative controls. The latter ones were constituted by isotype controls and the omission of the primary antibody. Also, the viral antigens were detected by an anti-DENV antibody that was kindly provided by the Evandro Chagas Institute, Para State, Brazil.

**Table 1:** Primary antibodies and detection systems in immunohistochemistry.

Antibody	Mark/code	Dilution	System of detection	Target
Monoclonal mouse anti-dengue	Evandro Chagas Institute/PA	1:100	Advance, Dako	Viral antigens
Polyclonal goat anti-RIG-I	Abcam/ab111037	1:100	Immpress, Vector	Molecular patterns in pathogens such as RNA in the cytoplasm. Plays a role in innate immune responses by triggering antiviral responses
Recombinant Monoclonal rabbit anti-STING	Abcam/ab227705	1:400	Immpress, Vector	The antibody targets STING, which is involved in immune responses, recruiting IRF3 to induce the secretion of type-1 IFN
Polyclonal rabbit anti-IRF2	Proteintech/125251AP	1:100	Ultravision, Thermo	IRF-2 is a transcription factor that binds to the promoter regions of type-I IFN and MHC class I genes
Monoclonal rabbit anti-IFN-I	Novus/NBP83119	1:200	Ultravision, Thermo	Interferon alpha/beta proteins, Both are key components of the type-I interferon response, which plays a crucial role in the defence against viral infections.

Immunolabeled cells were quantified in 10 randomized fields considering the midzonal region of the hepatic parenchyma. Positivity in portal space was also considered. The results are shown as number of cells/mm^2^. The comparison of the number of positive cells/mm^2^ was statistically analysed by the Mann–Whitney test under a 95% significance level (p < 0.05).

### Ethics

This research was approved by the Ethics and Research Committee of Medical School, Hospital das Clinicas under reference n° 253/12 in accordance with the Brazilian National Health Council (Resolution n° 466/12).

## RESULTS

According to autopsy data, the cases in this study involved 20 patients aged from 19 to 62 years, except for three children aged four, nine, and 10 years. These cases rapidly progressed to severe dengue with the involvement of various organs, showing macroscopic changes, primarily in the liver, lungs, kidneys, and the central nervous system. All patients showed positive dengue serology (IgM and IgG). The performed serology tests for hepatitis and leptospirosis served to discard such infections. Table 2 details the demographic, clinical, and histological analysis in this study.

**Table 2:** Demographic data from patients and histopathological evaluation of liver specimens.

Patient	Age	Gender	Dengue serology	Congestion	Necrosis in midzonal region	Necrosis in zone 3	Apoptosis	Lymphocytes	Macrophages	Neutrophils	Steatosis	Portal inflammation (lymphocytes)	Ductal reaction
1	09	M	+	2	0	0	1	1	1	0	0	1	0
2	19	F	+	3	2	1	1	1	1	0	0	1	0
3	52	M	+	1	0	1	2	2	1	2	0	2	1
4	04	M	+	1	0	0	1	1	0	0	0	0	0
5	30	F	+	1	3	3	0	0	0	0	0	1	0
6	52	F	+	1	0	0	1	2	1	1	20%	2	0
7	38	M	+	1	1	0	1	1	1	1	40%	1	0
8	41	F	+	1	0	0	1	1	1	1	80%	2	2
9	36	F	+	3	0	0	1	0	0	0	30%	1	0
10	25	F	+	0	0	0	2	3	1	0	0%	0	0
11	62	M	+	0	0	0	0	2	1	1	10%	2	0
12	28	F	+	3	2	0	1	1	1	1	50%	1	0
13	43	F	+	1	0	0	1	2	1	1	70%	1	0
14	37	M	+	3	3	3	0	0	0	0	0%	0	0
15	10	M	+	2	0	0	1	2	1	1	0%	1	0
16	41	F	+	1	3	2	1	2	1	2	0%	1	0
17	47	F	+	1	2	3	1	1	1	1	0%	1	0
18	26	M	+	1	0	0	1	1	0	0	0%	1	0
19	35	F	+	1	0	0	1	1	1	1	50%	1	0
20	37	M	+	2	1	1	2	0	0	0	20%	2	0

Score determined as absent (0), discrete (1), moderate (2), and intense (3).

**Figure 1:**
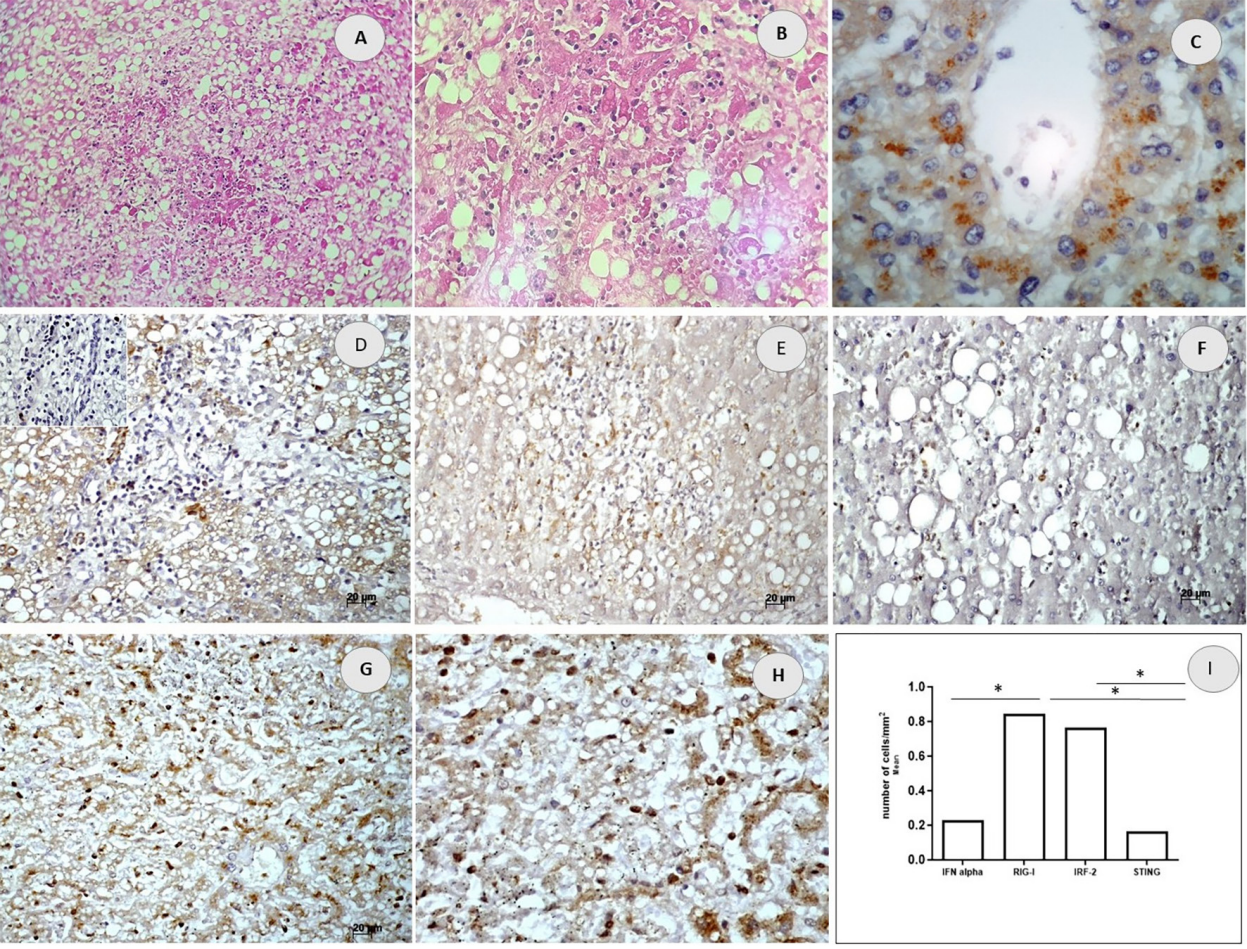
Haemorrhagic dengue, liver specimens: (A and B) Inflammatory infiltrate constituted by lymphocytes, macrophages, and neutrophils and foci of steatosis and necrosis (Haematoxylin-Eosin, x200 and x400); (C) Immunohistochemical detection of viral antigens (in brown) x400; (D-H) Immunohistochemical detection (brown) of the nuclear expression of IFN-α/β (D), RIG1 (E), STING (F) and IRF2 (G,H); (I) Quantitative and statistical analysis of cells expressing markers, Mann-Whitney test. Mean of positive cells (*p < 0.05).

The histological analysis of hepatic lesions evinced lobular necrosis in the midzonal region of the hepatic parenchyma in 50% of specimens. Apoptosis and portal inflammation occurred in 65% of samples. Inflammatory infiltration consisted of lymphocytes, macrophages, and neutrophils. This study also observed steatosis (Figures 1A and 1B).

All specimens showed DENV antigens in the cytoplasm of their mononuclear cells in the peri-portal region and inflammatory infiltrate of the hepatic parenchyma (Figure 1C). Immunohistochemical analysis of the innate immune factors evinced mononuclear cells expressing RIG1, IRF2, IFN- α/β, and STING in the nucleus or the perinuclear region (Figures 1D-1H). The quantification of such elements evinced an increased number of cells expressing RIG 1 and IRF 2 over STING and IFN-α/β (Figure 1I).

When comparing the expression of IFN- α/β (0.2208 cells/mm^2^; SD±0.599) to that of IRF-2 (0.7647 cells/mm^2^; SD±1.405) or to that of STING (0.1625 cells/mm^2^; SD±0.457) we found no statistical difference (p > 0.05). The analysis of cells expressing RIG-1 (0.8444 cells/mm^2^; SD±1.113) evinced a statistical difference when compared to IFN-α/β (p=0.008). Cells expressing RIG-1 did not differ when compared to IRF-2. IRF-2 and RIG-1 showed higher expressions than that of STING (p<0.05).

The analysis of portal space in the 20 specimens found cells expressing IFN-α/β in five cases, RIG 1 in four, IRF-2 in six, and STING in three.

## DISCUSSION

Plasma leakage occurs in the severe phase of dengue, increasing the risk of shock, haemorrhages, and dysfunctions in vital organs, such as the liver. To understand the action of the virus in this organ, this study used immunohistochemistry and histopathological analyses to correlate its results with hepatic lesions. Its findings included congestion, predominant necrosis in areas close to central zones (middle zone – Z2), haemorrhagic foci, steatosis, and hepatitis. These changes have been observed in post-mortem studies with the same technique^20–23^.

The histological evaluation of the liver samples found inflammatory cells (such as lymphocytes, histiocytes, and intralobular neutrophils) and lymphocytes in the portal space. These findings agree with the analyses performed in the autopsies, which also found these cell types in regions such as the sinusoids of the hepatic lobules and in the portal areas^24,25^.

Although necrosis configured the predominant finding (as in the results section), this study observed hepatocytes with signs of apoptosis in some areas. The viral pathogenesis of dengue in the liver has been described as cellular morphological changes compatible with anoxia and cell death by apoptosis^23^.

STING, although more commonly associated with double-stranded DNA viruses, may constitute a target of the dengue virus during infections. DENV can cleave the human STING protein, facilitating its invasion. Regarding RIG-I, a helicase that recognizes RNA viruses, some studies have indicated that its function in response to DENV fails to necessarily correlate with that of MDA5 to trigger a response. Moreover, that interferon factors participate in the type I interferon activation pathways stimulated by pattern recognition receptors. Among them, the regulatory factors IRF-1 and IRF-2 stand out for their importance in modulating this response. Due to the well-established role of IRF-1, we chose to more greatly emphasize IRF-2, which acts in an opposing manner by regulating interferon production and preventing excessive responses, especially in more severe infections, such as acute cases of dengue^26,27^.

Regarding pattern recognition receptors, the dengue virus uses several mechanisms to interfere with cell signalling pathways, consequently compromising IFN-I induction. The averages observed for the markers STING and IFN-I can be attributed to the ability of the virus to negatively modulate these pathways by non-structural proteins. The NS3 protein, for example, inhibits the translocation of RIG-I to mitochondria and can specifically cleave the STING protein in human cells, affecting the induction of interferon-I^28,29^.

This cleavage may justify the found lower levels of STING than of RIG-I since its degradation directly compromises interferon signalling mediated by this marker.

DENV infections activate RIG-I in association with MDA5 and TLR3, which play an important function for hosts’ defence against the virus^30^.

RIG-I induces several antiviral factors that can reduce the possible development of antiviral resistance (which have been described in dengue and Chikungunya^31^). On the other hand, RIG-I activation mediated by DENV may also play a role in pathogenesis by modulating cytokine production^32^.

Finally, it was interesting to observe a high number of cells expressing IRF-2. This factor frequently plays an antagonistic role in immune regulation, inhibiting the activation of IFN-I genes via competitive binding to shared sites, predominantly acting as a suppressor of IRF1-mediated gene activation^7^.

Despite having no statistically significant differences between IRF-2 and IFN-I, the higher mean of IRF-2 can be explained by its regulatory function, which modulates IFN-I induction. At the beginning of the infectious process, an exacerbated induction of IFN-I may have occurred, which could justify the increase in IRF-2 at the end of the disease. However, a more in-depth study of this marker is required to better understand this phenomenon^33,34^.

It is also important to mention that the mechanisms of innate immune evasion, such as IFN-I evasion, can play an important role in viral pathogenesis. Moreover, DENV can limit the production of IFN-I, blocking the signal induced by this cytokine in infected cells^35^.

Finally, we can consider some possible limitation during the quantification of the results in this study. The quantification in the midzonal region occurred by evaluating the identification of immunolabeled cells, which may vary according to immunostaining intensity. However, to minimize potential sources of bias, this study carried out the immunostaining for each antibody at the same moment, ensuring the homogeneity of staining intensity across results. Possible variations could arise from pre-analytical factors, which this study minimized during tissue preparation prior to antibody application. In this research, two observers performed the evaluations, comparing their results (which showed homogeneity, with no differences that could introduce uncertainty in their interpretation).

## CONCLUSION

Collectively, our findings indicate that in severe cases of dengue, the virus can evade effective immune responses by impairing the early activation of innate immunity. This immune dysregulation may contribute to the progression of more severe clinical manifestations. It also seems to play a role in the pathogenesis of hepatic involvement.

## Data Availability

The anonymized dataset generated during this study is available from the corresponding author upon reasonable request.

## References

[1] Pan American Health Organization (2025). Dengue epidemiological situation in the region of the Americas: epidemiological week.

[2] World Health Organization Global dengue surveillance.

[3] Brasil (2025). Ministério da Saúde. Secretaria de Vigilância em Saúde e Ambiente. Departamento de Doenças Transmissíveis.

[4] Kumar N, Gadpayle A, Trisal D (2013). Atypical respiratory complications of dengue fever. Asian Pac J Trop Dis.

[5] Fonseca BA, Fonseca SN (2002). Dengue virus infections. Curr Opin Pediatr.

[6] Huerre MR, Lan NT, Marianneau P, Hue NB, Khun H, Hung NT (2001). Liver histopathology and biological correlates in five cases of fatal dengue fever in Vietnamese children. Virchows Archiv.

[7] Castillo Ramirez JA, Urcuqui-Inchima S (2015). Dengue virus control of type I IFN responses: a history of manipulation and control. J Interferon Cytokine Res.

[8] Huang J, Liang W, Chen S, Zhu Y, Chen H, Ka C (2018). Serum cytokine profiles in patients with dengue fever at the acute infection phase. Dis Markers.

[9] Talarico LB, Byrne AB, Amarilla S, Lovera D, Vázquez C, Chamorro G (2017). Characterization of type I interferon responses in dengue and severe dengue in children in Paraguay. J Clin Virol.

[10] De La Cruz Hernández SI, Puerta-Guardo H, Flores-Aguilar H, González-Mateos S, López-Martinez I, Ortiz-Navarrete V (2014). A strong interferon response correlates with a milder dengue clinical condition. J Clin Virol.

[11] Pech Torres RE, Cedillo Rivera RM, Loroño Pino MA, Sánchez Burgos GG (2015). Serum levels of IFN-β are associated with days of evolution but not with severity of dengue. J Med Virol.

[12] Carlin AF, Plummer EM, Vizcarra EA, Sheets N, Joo Y, Tang W (2017). An IRF-3-, IRF-5-, and IRF-7-independent pathway of dengue viral resistance utilizes IRF-1 to stimulate type I and II interferon responses. Cell Rep.

[13] Schoggins JW, Dorner M, Feulner M, Imanaka N, Murphy MY, Ploss A (2012). Dengue reporter viruses reveal viral dynamics in interferon receptor-deficient mice and sensitivity to interferon effectors in vitro. Proc Natl Acad Sci U S A.

[14] Brass AL, Huang IC, Benita Y, John SP, Krishnan MN, Feeley EM (2009). The IFITM proteins mediate cellular resistance to influenza A H1N1 virus, West Nile virus, and dengue virus. Cell.

[15] Muñoz-Jordán JL, Laurent-Rolle M, Ashour J, Martínez-Sobrido L, Ashok M, Lipkin WI (2005). Inhibition of alpha/beta interferon signaling by the NS4B protein of flaviviruses. J Virol.

[16] Ashour J, Laurent-Rolle M, Shi PY, García-Sastre A (2009). NS5 of dengue virus mediates STAT2 binding and degradation. J Virol.

[17] Morrison J, Laurent-Rolle M, Maestre AM, Rajsbaum R, Pisanelli G, Simon V (2013). Dengue virus co-opts UBR4 to degrade STAT2 and antagonize type I interferon signaling. PLoS Pathog.

[18] Shen TJ, Chen CL, Jhan MK, Tseng PC, Lin CF (2020). CNS immune profiling in a dengue virus-infected immunocompetent outbred ICR mice strain. Front Cell Infect Microbiol.

[19] Devarbhavi H, Ganga D, Menon M, Kothari K, Singh R (2020). Dengue hepatitis with acute liver failure: clinical, biochemical, histopathological characteristics and predictors of outcome. J Gastroenterol Hepatol.

[20] Verdeal JC, Costa R, Vanzillotta C, Macedo GL, Bozza FA, Toscano L (2011). Guidelines for the management of patients with severe forms of dengue. Rev Bras Ter Intensiva.

[21] Burke T (1968). Dengue haemorrhagic fever: a pathological study. Trans R Soc Trop Med Hyg.

[22] Malavige GN, Ogg GS (2024). Molecular mechanisms in the pathogenesis of dengue infections. Trends Mol Med.

[23] Seneviratne SL, Malavige GN, Silva HJ (2006). Pathogenesis of liver involvement during dengue viral infections. Trans R Soc Trop Med Hyg.

[24] Bhamarapravati N, Tuchinda P, Boonyapaknavik V (1967). Pathology of Thailand haemorrhagic fever: a study of 100 autopsy cases. Ann Trop Med Parasitol.

[25] Couvelard A, Marianneau P, Bedel C, Drouet MT, Vachon F, Hénin D (1999). Report of a fatal case of dengue infection with hepatitis: demonstration of dengue antigens in hepatocytes and liver apoptosis. Hum Pathol.

[26] Maringer K, Fernandez-Sesma A (2014). Message in a bottle: lessons learned from antagonism of STING signalling during RNA virus infection. Cytokine Growth Factor Rev.

[27] Mamane Y, Heylbroeck C, Génin P, Algarté M, Servant MJ, LePage C (1999). Interferon regulatory factors: the next generation. Gene.

[28] Dalrymple NA, Cimica V, Mackow ER (2015). Dengue virus NS proteins inhibit RIG-I/MAVS signaling by blocking TBK1/IRF3 phosphorylation: dengue virus serotype 1 NS4A is a unique interferon-regulating virulence determinant. mBio.

[29] Liu H, Zhang L, Sun J, Chen W, Li S, Wang Q (2017). Endoplasmic reticulum protein SCAP inhibits dengue virus NS2B3 protease by suppressing its K27-linked polyubiquitylation. J Virol.

[30] Nasirudeen AM, Wong HH, Thien P, Xu S, Lam KP, Liu DX (2011). RIG-I, MDA5 and TLR3 synergistically play an important role in restriction of dengue virus infection. PLoS Negl Trop Dis.

[31] Olagnier D, Scholte FE, Chiang C, Albulescu IC, Nichols C, He Z (2014). Inhibition of dengue and Chikungunya virus infections by RIG-I-mediated type I interferon-independent stimulation of the innate antiviral response. J Virol.

[32] Conceição TM, Rust NM, Berbel AC, Martins NB, Santos CA, Poian AT (2012). Essential role of RIG-I in the activation of endothelial cells by dengue virus. Virology.

[33] Matveeva OV, Chumakov PM (2018). Defects in interferon pathways as potential biomarkers of sensitivity to oncolytic viruses. Rev Med Virol.

[34] Taniguchi T, Ogasawara K, Takaoka A, Tanaka N (2001). IRF family of transcription factors as regulators of host defense. Ann Rev Immunol.

[35] Muñoz-Jordán JL, Fredericksen BL (2010). How flaviviruses activate and suppress the interferon response. Viruses.

